# Driving status and health-related quality of life among the oldest old: a population-based examination using data from the AgeCoDe–AgeQualiDe prospective cohort study

**DOI:** 10.1007/s40520-020-01482-7

**Published:** 2020-01-31

**Authors:** André Hajek, Christian Brettschneider, Dagmar Lühmann, Hendrik van den Bussche, Birgitt Wiese, Silke Mamone, Siegfried Weyerer, Jochen Werle, Verena Leve, Angela Fuchs, Susanne Röhr, Janine Stein, Horst Bickel, Edelgard Mösch, Kathrin Heser, Michael Wagner, Martin Scherer, Wolfgang Maier, Steffi G. Riedel-Heller, Michael Pentzek, Hans-Helmut König

**Affiliations:** 1 Department of Health Economics and Health Services Research, Hamburg Center for Health Economics, University Medical Center Hamburg-Eppendorf, Hamburg, Germany; 2 Department of Primary Medical Care, Center for Psychosocial Medicine, University Medical Center Hamburg-Eppendorf, Hamburg, Germany; 3 Institute of General Practice, Hannover Medical School, Hannover, Germany; 4 Medical Faculty Mannheim, Central Institute of Mental Health, Heidelberg University, Mannheim, Germany; 5 Institute of General Practice, Medical Faculty, Heinrich-Heine-University Düsseldorf, Düsseldorf, Germany; 6 Institute of Social Medicine, Occupational Health and Public Health, University of Leipzig, Leipzig, Germany; 7 Department of Psychiatry, Technical University of Munich, Munich, Germany; 8 Department of Neurodegenerative Diseases and Geriatric Psychiatry, University of Bonn, Bonn, Germany; 9 German Center for Neurodegenerative Diseases (DZNE), Bonn, Germany

**Keywords:** Driving habits, Automobile driving, Health-related quality of life, Cohort study, EQ-5D, Subjective well-being

## Abstract

**Background:**

It is almost unknown whether the driving status is associated with HRQOL among individuals in highest age.

**Aims:**

Based on a multicenter prospective cohort study, the objective of this study was to examine whether the driving status is associated with health-related quality of life (HRQOL) among the oldest old in Germany.

**Methods:**

Cross-sectional data from follow-up wave 9 (*n* = 544) were derived from the “Study on Needs, health service use, costs and health-related quality of life in a large sample of oldest-old primary care patients (85+)” (AgeQualiDe). Average age was 90.3 years (± 2.7; 86 to 101 years). The current driver status (no; yes) was used in our analysis. The EuroQoL EQ-5D questionnaire was used to assess HRQOL in this study.

**Results:**

Regression analysis showed that being a current driver was associated with the absence of problems in ‘self-care’ [OR 0.41 (95%-CI 0.17 to 0.98)], and ‘usual activities’ [OR 0.48 (0.26 to 0.90)], whereas it was not significantly associated with problems in ‘pain/discomfort’ [OR  0.82 (0.47 to 1.45)] and ‘anxiety/depression’ [OR  0.71 (0.36 to 1.39)]. Being a current driver was marginally significantly associated with the absence of problems in ‘mobility’ [OR 0.60 (0.34 to 1.06)]. While being a current driver was not associated with the EQ-VAS in the main model, it was positively associated with the driving status (*β* = 5.00, *p* < .05) when functional impairment was removed from the main model.

**Discussion:**

Our findings provide first evidence for an association between driving status and HRQOL among the oldest old.

**Conclusions:**

Future longitudinal studies are required to evaluate a possible causal relationship between driving status and HRQOL in very old individuals.

## Introduction

It is expected that changes in the demographic composition, i.e., a rise in the number of individuals in highest age in the upcoming decades, will occur. Along with this development, the quantity of older people having a driving license will also increase [1].

Generally, various studies have investigated the determinants of health-related quality of life (HRQOL) among individuals in old age [2–4]. Studies also exist investigating the association between HRQOL and driving restrictions in late life [5]. However, it is almost unknown whether the driving status is associated with HRQOL among individuals in *highest age* (often used synonymously to oldest old; to denote those aged 85 years and over) [6]. Thus far, one study (*n* = 126 patients with age-related maculopathy who were in a low-vision clinic during the past year) showed that driving status was related to *vision-specific* HRQOL (using the National Eye Institute Visual Function Questionnaire). In this study, non-drivers (*n* = 96) were on average 80 years (± 7 years) and drivers (*n* = 30) were on average 76 ± 7 years. To close this gap in knowledge, the purpose of the current study was to examine the association between the driving status and HRQOL among the oldest old based on a multicenter prospective cohort study.

According to the OECD [7], in Europe, the percentage of licensed drivers among individuals aged 65–74 years vary from 71 to 93% in men and 7 to 46% in women. A car enables mobility, autonomy, and social participation [8]. Particularly among the oldest old, driving a car might also be associated with factors such as access to goods and services, out-of-home activity, and maintaining social relationships [9, 10]. Furthermore, it has been shown that driving in old age is, among others, associated with a reduced likelihood of depression, lower levels of loneliness, and perceived freedom [11–16]. Consequently, we assume that being a current driver is associated with higher HRQOL.

## Methods

### Sample

This study is a cross-sectional analysis of the driving status and HRQOL. It was performed within the “Study on Needs, health service use, costs and health-related quality of life in a large sample of oldest-old primary care patients (85+)” (AgeQualiDe). The AgeQualiDe study is a large multicenter prospective cohort study which continues and extends the German Study on Ageing, Cognition and Dementia in Primary Care Patients (AgeCoDe) which started in 2003/2004 (*n* = 3327). Individuals were recruited via general practitioners (GP) offices at six centers (Bonn, Düsseldorf, Hamburg, Leipzig, Mannheim, Munich). Inclusion criteria at baseline were age 75 years and over, absence of dementia in the view of the GP, and at least one contact with the GP during the preceding 12 months. Exclusion criteria at recruitment were being an irregular patient of the participating practice, consultations only via home visits, residents of a nursing home, severe illness the GP would deem fatal within 3 months, insufficient German language skills, blind or deaf, and lack of ability to provide informed consent. Face-to-face interviews were conducted with trained staff (mainly physicians and psychologists). With the aim of standardization, the interviewers were trained (including a theoretically grounded instruction, coaching, supervised practice, and ongoing supervision) to conduct interviews via members of the study team.

Further details have been published elsewhere [17]. Among the 3327 individuals, *n* = 544 individuals participated in FU wave 9 and provided data on both driving behavior and HRQOL. The most important reasons for drop off were that patients died or refused participation.

The AgeCoDe as well as the AgeQualiDe study have been approved by the ethics committees of all participating study centers and comply with the ethical standards of the Declaration of Helsinki. Prior to participation, all participants gave written informed consent.

### Outcome measures: HRQOL

The EQ-5D-3L questionnaire covers five items referring to current problems in the dimensions “mobility,” “self-care,” “usual activities,” “pain/discomfort,” and “anxiety/depression” (in each dimension: no problems; moderate problems; extreme problems). Because the number of respondents reporting extreme problems was rather low in all EQ-5D dimensions, these five outcome measures were dichotomized (0 = no problems; 1 = moderate/extreme problems). Moreover, the visual analog scale (EQ-VAS) was used as outcome measure (ranging from 0 = worst imaginable health state to 100 = best imaginable health state). In total, six outcome measures were used.

### Independent variables

Individuals were asked about whether they currently drive a car (no; yes) [18]. In regression models, it was adjusted for sex, age, family status [married vs. other (widowed; divorced; single)] and educational level which was measured using the Comparative Analysis of Social Mobility in Industrial Nations (CASMIN) [19] classification (low education; middle education; high education).

The six item version of the Lubben Social Network Scale (LSNS) which demonstrated favorable psychometric properties was used to measure social network/social support [20]. Higher values (0–30) correspond to higher social network/social support. In our study, Cronbach’s alpha was 0.74. Furthermore, cognitive and functional impairments were included in our regression model. Rather, complex instrumental activities of daily living were quantified using the Lawton and Brody scale (from 0 = worst score to 8 = best score) [21]. The Global Deterioration Scale [22] (from 1 = no cognitive impairment to 7 = severe cognitive impairment) was used to assess cognitive impairment.

### Statistical analysis

First, stratified by problems (no problems; moderate/extreme problems) in the EQ-5D dimensions “mobility,” “self-care,” “usual activities,” “pain/discomfort,” and “anxiety/depression”, the driving status was described. Second, adjusting for various potential confounders, the association between the driving status and the EQ-5D dimensions were analyzed using logistic regressions (no problems; moderate/extreme problems). Moreover, multiple linear regressions were used to analyze the association between the driving status and the EQ-VAS. The level of significance was set at *α* = 0.05 and marginally significance was defined by 0.05 < *p* < 0.10. Statistical analysis was conducted using Stata Release 15.1 (Stata Corp., College Station, Texas).

## Results

### Sample characteristics and bivariate associations

Table 1 shows the driving status (stratified by the EQ-5D dimensions; *n* = 544). In total, 68.6% were female and average age was 90.3 years (± 2.7, 86–101).

**Table 1:** Descriptive statistics and bivariate associations (by EQ-5D dimensions; *n* = 544)

	Mobility		Self-care		Usual activities		Pain/discomfort		Anxiety/depression	
	No problems	Moderate/extreme problems	No problems	Moderate/extreme problems	No problems	Moderate/extreme problems	No problems	Moderate/extreme problems	No problems	Moderate/extreme problems
Driving a car										
YES: *N* (%)	42 (26.3%)	45 (11.7%)***	79 (22.0%)	8 (4.3%)***	68 (25.2%)	19 (6.9%)***	32 (20.8%)	55 (14.1%)^+^	73 (18.6%)	14 (9.3%)**
No: *N* (%)	118 (73.7%)	339 (88.3%)	280 (78.0%)	177 (95.7%)	202 (74.8%)	255 (93.1%)	122 (79.2%)	335 (85.9%)	320 (81.4%)	137 (90.7%)

Column percentages are reported. *P* values are based on* t* tests or Chi-square tests, as appropriate

***p < 0.001, **p < 0.01, *p < 0.05, ^+^*p* < 0.10

In unadjusted analysis, driving a car was significantly associated with lower probability of moderate/extreme problems in the EQ-5D dimensions mobility, self-care, usual activities, and anxiety/depression. The association between driving a car and the absence of problems in pain/discomfort was marginally significant (*p* < 0.10).

As regards the EQ-VAS, higher among car drivers (69.0 ± 19.6) reported a higher  score (p < .001) compared to non-drivers (60.7 ± 18.5) in unadjusted analysis. It is worth noting that these significant differences were present in both genders.

### Regression analysis

Prior to regression analysis, it was checked whether multicollinearity is a threat to the regression results. Therefore, variance inflation factors (VIFs) were calculated. However, VIFs were rather small (highest VIF was 1.57 and mean VIF was 1.29) which indicates that multicollinearity is not a threat to our findings. Results of multiple logistic regressions [outcome measures: EQ-5D dimensions (dichotomized)] are depicted in Table 2. Being a current driver was significantly associated with the absence of problems in ‘self-care’ [OR 0.41 (95%-CI 0.17–0.98)], and the absence of problems in ‘usual activities’ [OR 0.48 (0.26–0.90)], whereas it was not significantly associated with problems in ‘pain/discomfort’ [OR 0.82 (0.47–1.45)] and ‘anxiety/depression’ [OR 0.71 (0.36–1.39)]. Being a current driver was marginally significantly associated with the absence of problems in ‘mobility’ [OR 0.60 (0.34–1.06)].

**Table 2:** Results of multiple logistic regressions with problems in EQ-5D dimensions used as dependent variables (FU wave 9)

Independent variables	(1)	(2)	(3)	(4)	(5)
	Mobility	Self-care	Usual activities	Pain/discomfort	Anxiety/depression
Being a current driver (ref.: not being a current driver)	0.60^+^	0.41*	0.48*	0.82	0.71
	(0.34–1.06)	(0.17–0.98)	(0.26–0.90)	(0.47–1.45)	(0.36–1.39)
Potential confounders	✔	✔	✔	✔	✔
Constant	1.10	0.35	0.30	383.85^+^	10.06
	(0.00–1897.78)	(0.00–627.10)	(0.00–267.99)	(0.42–348116.32)	(0.01–9160.51)
Observations	543	543	543	543	543
Pseudo *R*^2^	0.12	0.28	0.18	0.04	0.04

Odds ratios were reported; 95% CI in parentheses

Potential confounders include: age, marital status, education, social network, function, and cognitive impairment

To quantify the level of education, the CASMIN classification was used. Lubben Social Network Scale ranges from 0 to 30, with higher values reflecting more social networks and more social support; Instrumental Activities of Daily Living Scale was used to quantify function, ranging from 0 (worst score) to 8 (best score); Global Deterioration Scale was used to quantify cognitive impairment, ranging from 1 (best score) to 7 (worst score)

***p < 0.001, **p < 0.01, *p < 0.05, ^+^*p* < 0.10

As regards control variables, being male was associated with the absence of problems in ‘pain/discomfort’ [OR 0.54 (0.32–0.88)] and ‘anxiety/depression’ [OR 0.56 (0.33–0.97)]. Age and marital status were not associated with any of the outcome measures. High education was associated with the absence of problems in ‘mobility’ [OR 0.54 (0.30–0.96)] and social network/social support was associated with the absence of problems in ‘anxiety/depression’ [OR 0.95 (0.91–0.99)]. Function was associated with the absence of problems in ‘mobility’ [OR 0.69 (0.61–0.78)], ‘self-care’ [OR 0.53 (0.46–0.60)], and ‘usual activities’ [OR 0.70 (0.62–0.78)]. Cognitive impairment was associated with the presence of problems in ‘usual activities’ [OR 1.28 (1.02–1.62)], whereas it was associated with the absence of problems in ‘pain/discomfort’ [OR: 0.67 (0.54–0.83)].

Results of multiple linear regressions (outcome measure: EQ-VAS) are depicted in Table 3. The outcome measure was not significantly associated with being a current driver (*β* = 3.62, *p* = 0.14). However, the outcome measure was significantly associated with higher function (*β* = 1.21, *p* < 0.01) and higher social network/social support (*β* = 0.45, *p* < 0.01), whereas it was not significantly associated with sex, age, marital status, educational level, and cognitive impairment.

**Table 3:** Results of multiple linear regression with EQ-VAS score used as dependent variable (FU wave 9)

Independent variables	EQ-VAS (with function as independent variable)	EQ-VAS (without function as independent variable)
Being a current driver (ref.: not being a current driver)	3.62	5.00*
	(2.47)	(2.45)
Potential confounders	✔	✔
Constant	70.57**	90.49***
	(26.40)	(25.25)
Observations	535	535
*R*^2^	0.09	0.08

Beta coefficients were reported; Cluster-robust standard errors in parentheses

Potential confounders include: age, marital status, education, social network, function (only in the first model), and cognitive impairment

To quantify the level of education, the CASMIN classification was used. Lubben Social Network Scale ranges from 0 to 30, with higher values reflecting more social networks and more social support; Instrumental Activities of Daily Living Scale was used to quantify function, ranging from 0 (worst score) to 8 (best score); Global Deterioration Scale was used to quantify cognitive impairment, ranging from 1 (best score) to 7 (worst score)

***p < 0.001, **p < 0.01, *p < 0.05, ^+^*p* < 0.10

In sensitivity analysis, functional impairment was removed from the main model, because it might be associated with driving status and the outcome measure. In this model, the EQ-VAS was positively associated with the driving status (*β* = 5.00, *p* < 0.05).

## Discussion

### Main findings

The purpose of this study was to investigate the association between the driving status and HRQOL among individuals in highest age using a multicenter prospective cohort study in Germany. Of all respondents, 71.6% reported problems in pain/discomfort, followed by mobility (70.7%), usual activities (50.6%), self-care (34.3%), and anxiety/depression (28.0%). Bivariate analysis revealed that driving a car was associated with lower probability of problems in all EQ-5D dimensions (except for pain/discomfort, *p* < 0.10). For example, the prevalence of problems in mobility was 51.7% among the car drivers (self-care: 9.2%; usual activities: 21.8%; pain/discomfort: 63.2%; anxiety/depression: 16.1%).

Regressions showed that while being a current driver was associated with the absence of problems in ‘self-care’, and ‘usual activities’, it was not significantly associated with problems in ‘pain/discomfort’ and ‘anxiety/depression’. Being a current driver was marginally significantly associated with the absence of problems in ‘mobility’. While being a current driver was not associated with the EQ-VAS in the main model, it was positively associated with the driving status when functional impairment was removed from the main model.

### Possible explanations and relation to previous studies

Our findings contribute to the existing evidence by demonstrating that driving a car is associated with increased HRQOL in *highest* age. Thus, it extends previous knowledge based on younger and/or small, geographically restricted samples.

The association between being a current driver and the absence of problems in ‘self-care’ appears very plausible. We strongly assume that this association might be explained by the fact that an individual gives up driving for safety reasons when he or she is not being able to take care for his or herself (washing or dressing his- or herself), because driving a car is a complex task involving different parts of the body (for example, cognitive, physical, or visual abilities). In accordance with this, it also appears plausible that being a current driver is associated with the absence of problems in ‘usual activities’. Unexpectedly, the association between being a current driver and the absence of problems in ‘mobility’ was only marginally significant. This might be explained by the fact that function was included in our main model. Actually, when function was removed from the main model, the association between being a current driver and the absence of problems in mobility was highly significant [OR 0.42 (0.24–0.73)]. This pattern also holds true for when EQ-VAS was used as outcome measure.

The driving status was not significantly associated with problems in ‘anxiety/depression’. This non-significant association is somewhat surprising given the fact that driving cessation was associated with an increase in depressive symptoms in other studies after adjusting for potential confounders [23, 24]. Stopping driving might reflect a decrease in autonomy, social relationships and independence. Therefore, one might conclude that not being a current driver is associated with problems in ‘anxiety/depression’. However, this association was only present in unadjusted analysis. After adjusting for various sociodemographic variables, social network/social support, as well as functional and cognitive health in our regression model, this association disappeared. This might explain why driving status was not associated with this outcome measure in our study.

Finally, it is conceivable to us that driving a car was not associated with problems in ‘pain/discomfort’ in multiple regression analysis. Problems in this dimension are commonly correlated with other sociodemographic or health-related factors (e.g., being female or cognitive dysfunction) in old age [25, 26], but not with driving status. Furthermore, we are also not aware of any studies reporting an association between driving status and problems in ‘pain/discomfort’. Future studies are also needed to clarify the directionality of the relationship between driving status and problems in ‘pain/discomfort’. Furthermore, it should be noted that in this sample of oldest old individuals, some of them are still driving with pain and anxiety/depression. Future research is needed to clarify whether this can have consequences for driving cessation and driving safety.

It is worth repeating and emphasizing that the AgeQualiDe study was conducted in six *large cities* in Germany. Generally, the local public transport is well developed in these cities. Thus, the importance of driving a car among the oldest old might be limited. This might explain why the driving status was only weakly associated with the outcome measures. Further research is required regarding the association between the driving status and HRQOL in rural areas where the public transport infrastructure is poorly developed compared to urban areas.

### Strengths and limitations

To the best of our knowledge, this study is one of the first studies to examine the association between the driving status and HRQOL among individuals in highest age using a multicenter prospective cohort study. In regression analysis, it was adjusted for several potential confounders such as cognitive impairment or functional impairment. Widely established instruments were used to measure these potential confounders. HRQOL was quantified using the widely used and validated EQ-5D instrument. As this study is cross-sectional, changes within individuals over time cannot be analyzed. Future studies could, for example, use recently developed doubly robust techniques (e.g., augmented inverse‐probability‐weighted estimators or inverse-probability-weighted regression adjustment approaches) to study the link between driving and quality of life.

It has been shown that some attrition bias is present in this study [27], suggesting that it might be difficult to generalize our findings to individuals with, for example, very severe cognitive impairment or extreme problems on the EQ-5D dimensions. As far as data are available, future studies should include the duration of driving cessation and their link with increased functional impairments. For example, the association between driving status and HRQOL may vary in strength between individuals immediately after a stroke with suddenly increased functional impairments and individuals with gradually increasing functional impairments.

## Conclusion

Our findings provide the first evidence for an association between driving status and HRQOL among the oldest old. Future longitudinal studies are required to evaluate a possible causal relationship between driving status and HRQOL in very old individuals. Furthermore, it might be interesting to know whether driving cessation is associated with the cognitive evaluation of life as a whole (life satisfaction).

In very recent years, a rapid increase in the number of power-assisted bicycles (so-called “pedelecs”) took place in Germany. Moreover, the number of intercity buses is steadily increasing in Germany. It might be worth investigating whether these factors affect the relation between driving status and HRQOL among the oldest old in the near future.
