# Correction to: Driving status and health-related quality of life among the oldest old: a population-based examination using data from the AgeCoDe–AgeQualiDe prospective cohort study

**DOI:** 10.1007/s40520-021-01920-0

**Published:** 2021-07-05

**Authors:** André Hajek, Christian Brettschneider, Dagmar Lühmann, Hendrik van den Bussche, Birgitt Wiese, Silke Mamone, Siegfried Weyerer, Jochen Werle, Verena Leve, Angela Fuchs, Susanne Röhr, Janine Stein, Horst Bickel, Edelgard Mösch, Kathrin Heser, Michael Wagner, Martin Scherer, Wolfgang Maier, Steffi G. Riedel-Heller, Michael Pentzek, Hans-Helmut König

**Affiliations:** 1 Department of Health Economics and Health Services Research, Hamburg Center for Health Economics, University Medical Center Hamburg-Eppendorf, Hamburg, Germany; 2 Department of Primary Medical Care, Center for Psychosocial Medicine, University Medical Center Hamburg-Eppendorf, Hamburg, Germany; 3 Institute of General Practice, Hannover Medical School, Hannover, Germany; 4 Medical Faculty Mannheim, Central Institute of Mental Health, Heidelberg University, Mannheim, Germany; 5 Institute of General Practice, Medical Faculty, Heinrich-Heine-University Düsseldorf, Düsseldorf, Germany; 6 Institute of Social Medicine, Occupational Health and Public Health, University of Leipzig, Leipzig, Germany; 7 Department of Psychiatry, Technical University of Munich, Munich, Germany; 8 Department of Neurodegenerative Diseases and Geriatric Psychiatry, University of Bonn, Bonn, Germany; 9 German Center for Neurodegenerative Diseases (DZNE), Bonn, Germany

## Correction to: Aging Clinical and Experimental Research https://doi.org/10.1007/s40520-020-01482-7

The article Driving status and health related quality of life among the oldest old: a population based examination using data from the AgeCoDe–AgeQualiDe prospective cohort study, written by André Hajek, Christian Brettschneider, Dagmar Lühmann, Hendrik van den Bussche, Birgitt Wiese, Silke Mamone, Siegfried Weyerer, Jochen Werle, Verena Leve, Angela Fuchs, Susanne Röhr, Janine Stein, Horst Bickel, Edelgard Mösch, Kathrin Heser, Michael Wagner, Martin Scherer, Wolfgang Maier, Steffi G. Riedel-Heller, Michael Pentzek and Hans-Helmut König was originally published electronically on the publisher’s internet portal (currently SpringerLink) on 11 rd January 2021 without open access. With the author(s)’ decision to opt for open choice, the copyright of the article changed on 1st July 2021 to © The Author(s) 2021 and the article is forthwith distributed under the terms of the Creative Commons Attribution 4.0 International License (http://creativecommons.org/licenses/by/4.0/), which permits use, duplication, adaptation, distribution and reproduction in any medium or format, as long as you give appropriate credit to the original author(s) and the source, provide a link to the Creative Commons license and indicate whether changes were made.

The original article has been corrected.

